# Efficacy and Safety of Intro-Arterial Chemotherapy Combined with Radiotherapy on Head and Neck Cancer: A Systematic Review and Meta-Analysis

**DOI:** 10.7150/jca.36478

**Published:** 2019-10-17

**Authors:** Yan Huang, Li-Ling Wu, Ruo-Lan Xiang, Guang-Yan Yu

**Affiliations:** 1 Department of Oral and Maxillofacial Surgery, Peking University School and Hospital of Stomatology, Beijing, 100081, China.; 2 Department of Physiology and Pathophysiology, Peking University School of Basic Medical Sciences, Key Laboratory of Molecular Cardiovascular Sciences, Ministry of Education, and Beijing Key Laboratory of Cardiovascular Receptors Research, Beijing, 100191, China.

**Keywords:** intro-arterial chemoradiotherapy, head and neck cancer, complete response, overall survival, toxicity

## Abstract

**Objectives**: Intro-arterial chemotherapy combined with radiotherapy (IACRT) for the treatment of head and neck cancer (HNC) underwent a revival in recent years. Although many clinical trials have reported favorable outcomes, the effect of IACRT for HNC is still controversial. Therefore, this study was designed to evaluate the efficacy and safety of IACRT for HNC.

**Methods**: The relevant articles published before August 2019 were searched from PubMed, Embase, Cochrane Library, Web of Science and PMC databases. Data were extracted and the combined complete response (CR), overall survival (OS) and toxicity incidence with 95% credible interval (CI) were examined from eligible studies.

**Results**: Thirty-four studies comprising 1890 patients were included. IACRT achieved high CR (0.81, 95% CI: 0.76-0.86, *P* < 0.001), 3-year OS (0.75, 95% CI: 0.68-0.82, *P* < 0.001) and 5-year OS (0.68, 95% CI: 0.61-0.75, *P* < 0.001). The 3-year OS rate of stage III cancer (0.75, 95% CI: 0.53-0.97, *P*< 0.001) was higher than stage IV (0.52, 95% CI: 0.37-0.66, *P* = 0.025). Meanwhile, the 5-year OS of T3 cancer (0.87, 95% CI: 0.73-1.01, *P* = 0.028) was higher than T4 (0.53, 95% CI: 0.42-0.63, *P* = 0.286). Additionally, oral diseases, mucositis, leukopenia and dermatitis were the major toxicities of IACRT, which were all reversible.

**Conclusion**: IACRT is an efficient and safe modality for HNC, which could achieve favorable cancer response and higher survival rate with acceptable toxicities, even for advanced HNC.

## Introduction

Head and neck cancer (HNC) is the 6th most common malignancy worldwide, most patients presented advanced stage when diagnosed, which was characterized by low 5-years survival rate and poor prognosis 1-3. The traditional treatments for HNC are surgery, radiotherapy and chemotherapy. Owing to the complexity of structure and the need for organ preservation, these traditional treatments for HNC have many limitations. Cancers with invasion to deep and important location or with multiple distant metastases are unresectable 4, 5. Moreover, surgical treatment would cause a significant decrease in physical function and cosmetic impairment. Radiation therapy often induces many adverse effects such as oral mucositis, osteoradionecrosis, tissue fibrosis and salivary gland dysfunction 6, 7. Therefore, novel therapies with stronger effect and lower toxicity are urgently needed.

Since most HNCs are insensitive to anticancer drugs, chemotherapy alone is not a preferred treatment for HNC 8. It is usually combined with radiation therapy. Early practice was mainly focus on intra-versus chemotherapy combined with radiotherapy (IVCRT), although there are certain advances by employing IVCRT, the results of HNC patients are still unsatisfactory, due to low drug level in cancer site and high drug level in circulatory system. In recent years, intra-arterial chemotherapy combined with radiotherapy (IACRT) for HNC attracts many attentions, which was proved to get favorable results in many clinical trials, especially after advanced angiographic techniques developed, permitting the infusion of drugs superselectively into tumor feeding arteries 9, 10. The greatest advantages of IACRT are increased drug concentration at the tumor bed and mild systemic toxicity due to relatively low level of the drug in blood circulation 11. Robbins and his co-workers inserted a catheter into femoral artery with cisplatin, which reported to achieve high percentage of local control without increasing adverse effects in advanced carcinoma of the head and neck 12, 13. Mitsudo et al. achieved high complete response (CR = 95.8%) and 3-year overall survival (OS = 81.5%) through using retrograde superselective IACRT for tongue cancer 14. Although many studies showed favorable outcomes, there are still some controversies. Some investigations only obtain poor CR rate (38.5%) and lower 5-year OS rate (39.5%) 15, 16. Therefore, the exact efficacy of IACRT remains controversial, especially for cancers in different stages, which may be more valuable for clinicians.

Therefore, this meta-analysis was conducted to evaluate the efficacy and safety of IACRT on the treatment of HNC patients. Furthermore, by dividing patients according to the stages, we attempted to analyze IACRT efficacy in different stage HNC, which may provide the summarized and evidence-based information for clinicians in treating HNC patients.

## Methods

This systematic review and meta-analysis was complied with PRISMA Statement 17.

### Search strategy

We manually searched and selected documents with the following search terms: “radiation therapy” and “intra-arterial chemotherapy” or “intra-arterial drug administration” and “mouth cancer” or “mouth carcinoma” or “tongue carcinoma” or “mouth tumor” or “salivary gland tumor” or “parotid gland tumor” or “tongue tumor” or “pharynx tumor” or “tonsil tumor” or “oral cancer” or “oral tumor” or “mouth squamous cell carcinoma” or “gingival carcinoma” or “carcinoma of gingiva” or “oropharynx carcinoma” or “head and neck cancer” or “head and neck tumor” from PubMed, Embase, Cochrane Library, Web of Science and PMC databases. Additionally, we search original published articles, reviews, references cited in the relevant review articles, conference abstracts and the publications of experts up to August 2019 to find the related articles.

### Study selection

All literatures were manually read and screened in dependently by two reviewers (Y. Huang and R.L. Xiang). Any discrepancies between the two reviewers were solved by a consensus. Animal studies and non-English articles were excluded. If the review includes raw data, detailed examination of the full text will lead the studies to be included or excluded. If there were duplicate studies, we choose articles published most recently or providing more detailed information. If review contains raw data, it will also be included.

### Data extraction and quality assessment

An investigator used a pre-designed structure table to extract data from each eligible study, another investigator independently reviewed the data to ensure accuracy. Eligible trials have to satisfy the Participants, Intervention, Comparison, Outcomes, and Setting (PICOS) criteria: (1) Participants: HNC patients, (2) Intervention: intra-arterial chemoradiotherapy, (3) Comparison: no comparison, (4) Outcomes: CR or OS of IACRT therapy, (5) Setting: most studies are retrospective studies. The studies of intra-arterial chemotherapy (IACT) without radiotherapy were not included. Additionally, we included one RCT and three prospective studies, but only data from the IACRT group were used.

Since most of the studies included were single-arm cohort studies, we used the CASP-Cohort-Study-Checklist to assess the quality of the studies. Two authors (Y. Huang and R.L. Xiang) assessed the quality and risk of bias in the eligible studies independently. Disagreements were solved through discussion and reevaluation.

### Statistical analysis

We evaluated therapeutic effect of IACRT in the treatment of HNC by assessing CR and OS with their 95% credible interval from included trials, and drawing plotted forest map according to the results. We also performed a subgroup analysis to assess the impact of IACRT to different stage HNCs. A combined analysis of OS in the graded patients was conducted including the 3-year OS of stage III and stage IV cancers as well as the 5-year OS of T3 and T4 cancers. Furthermore, we calculated toxicity incidence with 95% credible interval of enrolled reports to assess the safety of IACRT. All statistical analyses were conducted by using Stata14 statistical software, and a *P* < 0.05 was considered to have statistical significance. *I^2^* statistics were used to measure statistical heterogeneity. If *I^2^* > 50%, a random-effect model was used, otherwise, a fixed-effect statistical model was used. Stata 14 Egger's test and Begg's funnel plot were used to assess publication bias, *P* value lower than 0.05 were judged as statistically significant, indicating that the study has publication bias.

## Results

### Eligible studies

**Figure 1:**
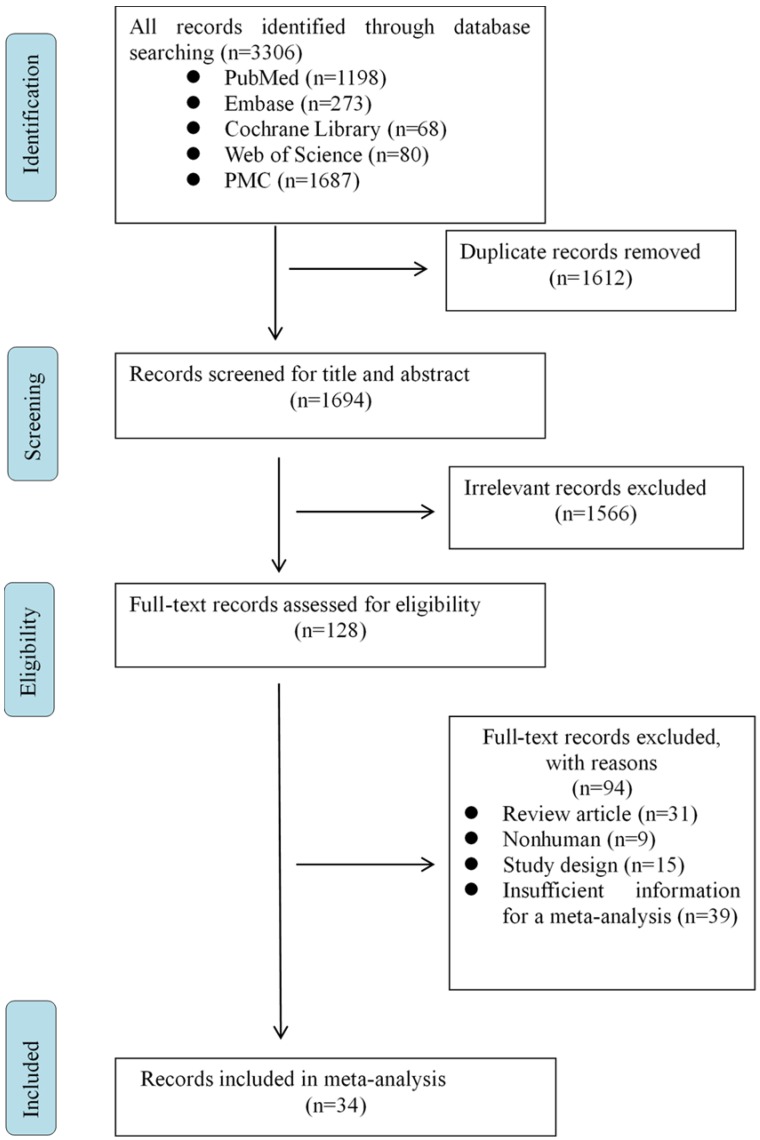
Flow program of eligible studies.

A search in PubMed, Embase, Cochrane Library, Web of Science and PMC databases yielded a total number of 3306 articles. After removing duplicated and irrelevant records, only 128 full-text articles remained for eligibility. We then excluded the following studies: review article, nonhuman study, study design, insufficient information for meta-analysis. Finally, 34 papers were identified for the present meta-analysis (Fig. 1).

### Study characteristics

The meta-analysis included 34 studies consisting with a total number of 1890 patients. Among which 27 studies were from Japan, 3 studies were from Italy, others were from USA, Korea, Germany and Macedonia, respectively. Ten types of HNCs were investigated in this meta-analysis, including squamous cell carcinoma (SCC) of tongue, gingiva, mouth floor, buccal, palate, larynx, oropharynx, maxillary sinus, paranasal sinus and nasal natural killer/T-cell lymphoma (NNKTL). Among these records, 30 were retrospective studies, 3 were prospective study, only one was randomized controlled trial (RCT). Moreover, 5 studies provided 5-year OS rate, 9 studies reported both 5-year OS and CR rates, 10 studies assessed both 3-year OS and CR values, and just 5 studies estimated all 3-year OS, 5-year OS and CR values. The detailed information of each study was listed in Table 1.

### Results of individual studies and data synthesis

#### Complete response (CR)

**Figure 2:**
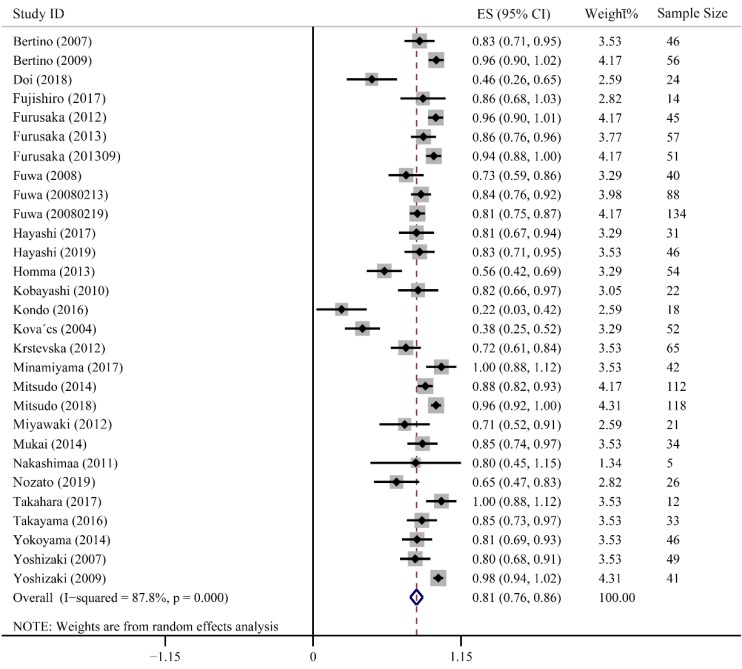
Forest plot of complete response.

CR refers complete clearance of the lesion after treatment, which directly reflects the clinic efficiency of cancer treatment. Twenty-nine studies containing 1382 patients were enrolled to evaluate CR value, in which, 24 studies presented high CR rates ranging from 0.71-1.00. Furusaka et al. achieved 96%, 86% and 94% CR rates in SCC of tongue, hypopharyngeal piriform sinus and anterior oropharyngeal wall, respectively 20-22. Additionally, 73% and 84% of CR rates were reported by Fuwa et al. using carboplatin and cisplatin, respectively 16, 23. Only 2 studies had relatively lower CR rates of 0.22 (95% CI: 0.03-0.42) and 0.38 (95% CI: 0.25-0.52). The combined CR value was up to 0.81 (95% CI: 0.76-0.86, *P* < 0.001). Heterogeneity in these studies was higher than 50% (*I^2^* = 87.8%), so we used a random-effect model (Fig. 2).

#### Overall survival (OS)

**Figure 3:**
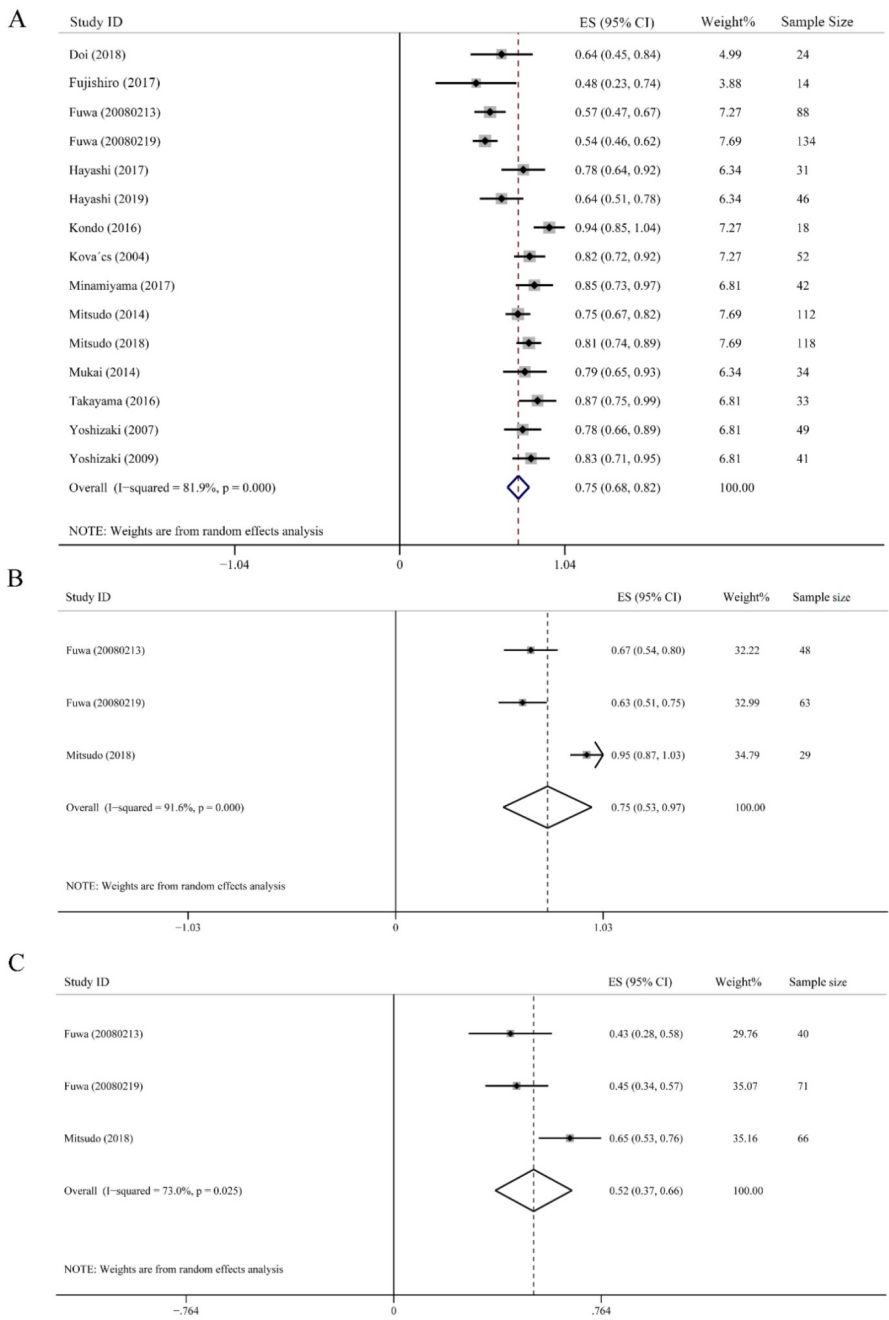
Forest plots of 3-year overall survival (OS). **(A)** 3-year OS of all HNC. **(B)** 3-year OS of stage III HNC. **(C)** 3-year OS of stage Ⅵ HNC.

OS was defined as the time from the commencement of treatment until death or last follow-up time, which largely represents treatment efficacy. The 3-year OS rate was calculated from 15 studies including 836 subjects. As showed in Fig. 3A, the highest 3-year OS was 0.94 (95% CI: 0.85-1.04), and the lowest one was 0.48 (95% CI: 0.23-0.74). Despite high heterogeneity (*I^2^* = 81.9%), the merged 3-year OS rate was still up to 0.75 (95% CI: 0.68-0.82, *P* < 0.001). Additionally, a random-effect model was employed (Fig. 3A). We further conducted subgroup analysis on the effect of IACRT on 3-year OS ratio of stage III and stage IV HNC. Data were extracted from 3 studies consisted of 140 patients in stage III and 177 patients in stage IV HNC, who underwent IACRT. The combined 3-year OS rate of stage III and stage IV were 0.75 (95% CI: 0.53-0.97, *P* < 0.001) and 0.52 (95% CI: 0.37-0.66, *P* = 0.025), respectively. The heterogeneity of stage III and stage IV HNC were 91.6% and 73.0%, respectively. Therefore, random-effect model was used (Figs. 3B and 3C).

**Figure 4:**
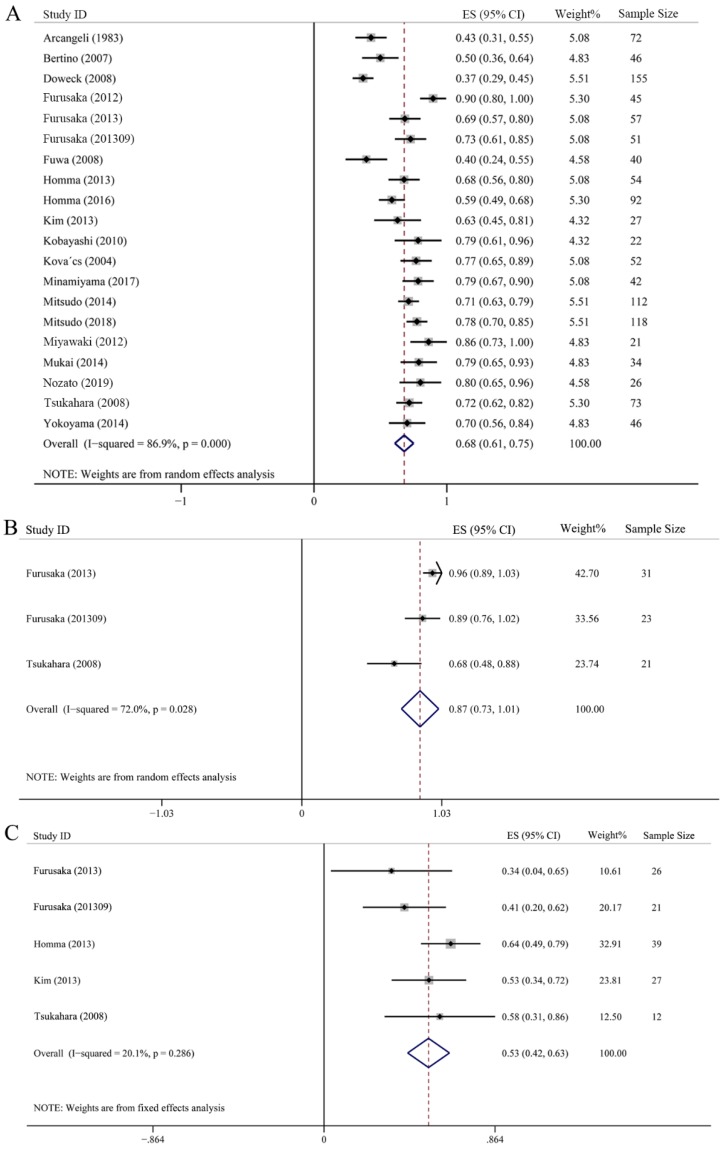
Forest plots of 5-year overall survival (OS). **(A)** 5-year OS of all HNC. **(B)** 5-year OS of T3 HNC. **(C)** 5-year OS of T4 HNC.

Twenty studies with 1185 samples were included in the 5-year OS assessment, among them, 5 studies also provided 3-year OS rate. As showed in Fig. 4A, the 5-year OS rate was scattered among 0.37-0.90, whose synthesis value was 0.68 (95% CI: 0.61-0.75, *P* < 0.001) just litter lower than that of 3-year OS, this indicated that there was no difference between 3-year OS and 5-year OS by using IACRT. In addition, we also analyzed the 5-year OS in T3 and T4 subgroups. Three studies with 75 patients and 5 studies with 125 patients were included in T3 and T4 group, respectively. It should be noted that some studies classified T4 stage more detailed including T4a and T4b. Therefore, we merged the OS ratio of T4a and T4b of these studies in advance, and then used the combined OS value to merge with other studies. The combined 5-year OS rate of T3 was 0.87 (95% CI: 0.73-1.01, *P* = 0.028), however, the 5-year OS rate was 0.53 (95% CI: 0.42-0.63, *P* = 0.286) in T4 subgroup, which was much lower than T3 stage. A random-effect model was adopted in T3 subgroup as *I^2^* = 72%. However, a fixed-effect statistical model was used in T4 subgroup for low heterogeneity (*I^2^* = 20.1%) (Figs. 4B and 4C).

#### Toxicity

**Figure 5:**
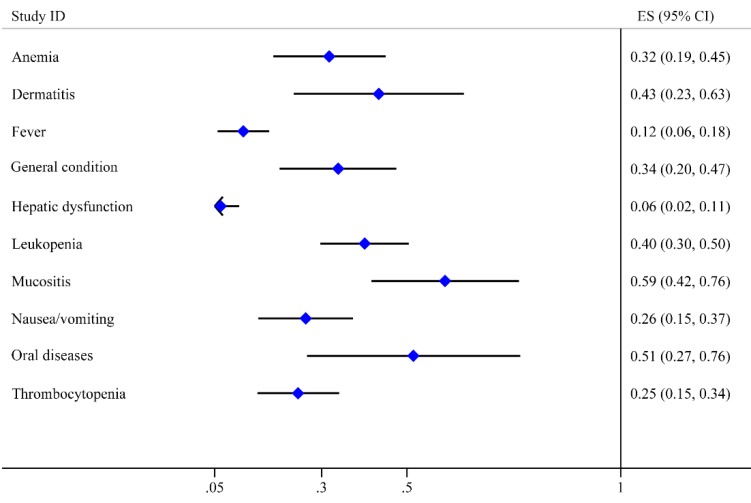
Forest plot of toxicities.

Toxicity can reflect the safety characteristic of treatment. It is regarded as an essential indicator of prognosis. Therefore, we extracted all side-effect data from 34 studies, which mentioned nearly 70 side-effect reactions. We reclassified and extracted 10 kinds of toxicities after excluding the side effects mentioned in less than three studies such as hyperuricemia, renal disorders, gastrointestinal diseases and osteoradionecrosis. Oral toxicities including oral diseases reported by 10 studies and mucositis reported by 21 studies were the major complications with the high incidence of 0.51 (95% CI: 0.27-0.76) and 0.59 (95% CI: 0.42-0.76), respectively. The next two toxicities were dermatitis (0.43, 95% CI: 0.23-0.63) and leukopenia (0.40, 95% CI: 0.30-0.50), The incidence rates of general condition, anemia, nausea/vomiting and thrombocytopenia were lower than 0.35. Fever and hepatic dysfunction were the last two toxicities with the lowest incidence rates of 0.12 (95% CI: 0.06-0.18) and 0.06 (95% CI: 0.02-0.11), respectively (Table 2). All these adverse effects were manageable and reversible. Moreover, we mapped the forest graph based on the incidence rates of these 10 toxicities (Fig. 5).

### Risk of bias across studies

**Figure 6:**
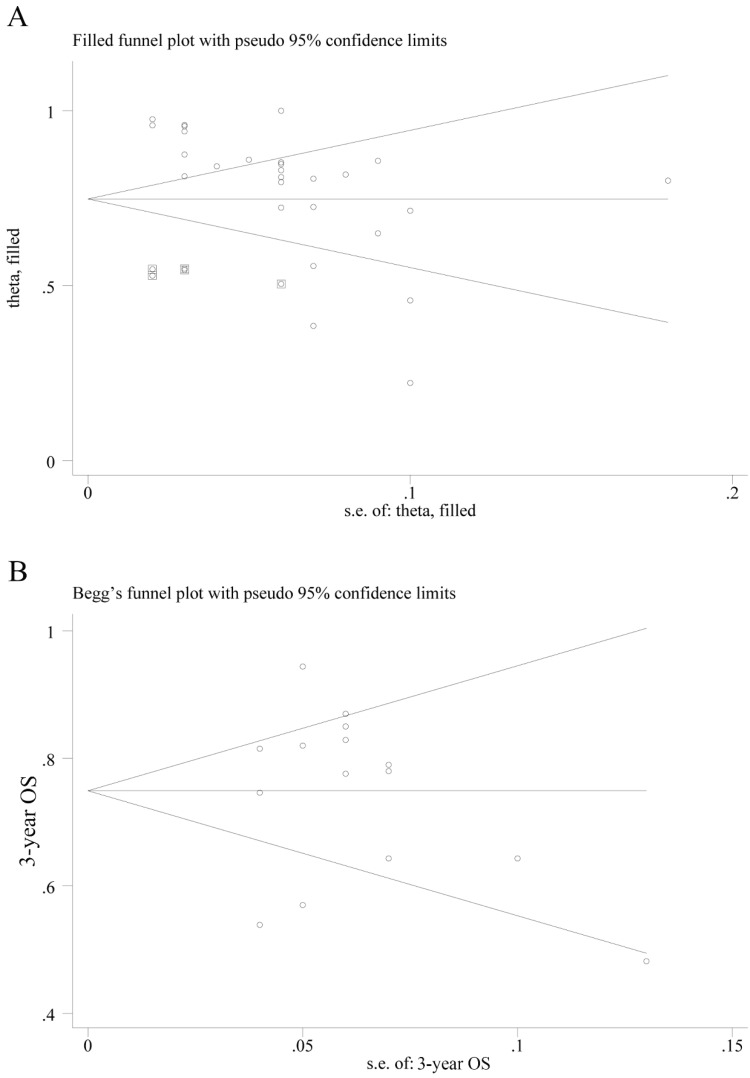
Publication bias on complete response (CR) and overall survival (OS). **(A)** Begg's funnel plot of the eligible studies involving CR combination; **(B)** Begg's funnel plot of the eligible studies involving 3-year OS.

The *P* value of Egger's test in CR combination was less than 0.001, indicating that significant publication bias exists. Therefore, we used trim-fill method and found that by filling 6 studies, the bias can be eliminated. The effect value of CR was 0.748 (95% CI: 0.680 - 0.816) (Fig. 6A). However, we haven't found suitable study yet. The Egger's test of 3-year OS showed no significant publication bias for *P* = 0.969, and the Begg's funnel plot was shown in Fig. 6B.

## Discussion

In the current systematic review and meta-analysis, 34 clinical trials including 1890 subjects diagnosed as HNC were enrolled. We assessed the efficacy of IACRT by calculating CR and OS rates in HNC patients underwent IACRT. The combined CR, 3-year OS and 5-year OS rates were all favorable. However, when assessing OS by classification of cancer into detail stages, the 3-year OS rate of stage III was higher than stage IV. Simultaneously, the 5-year OS rate of T3 patients was much higher than T4 patients. Additionally, oral diseases, mucositis, leukopenia and dermatitis were the common but reversible toxicities of IACRT. Therefore, we concluded that IACRT is efficacious for HNC which not only increases patient's survival rate, but also improves patient's quality of life.

In recent decades, IACRT was introduced to improve the outcome of patients with advanced HNC. Compared to conventional therapy, it is a friendly approach for preservation of organ and its function. Moreover, it can increase tumor responsiveness by increasing drug concentrations at the tumor site, meanwhile minimize the dose-limiting systemic side effects of drugs 48-50. Concurrent combination of arterial infusion therapy with high dose of cisplatin and radiotherapy achieved 80% of CR in T3 and T4 HNC patients, which was only 61% in systemic chemotherapy combined with radiotherapy 12, 13. Another study analyzed the therapeutic results of retrograde superselective intra-arterial chemoradiotherapy in 118 patients with tongue cancer, and got a CR of 0.96 and a 3-year OS of 0.82, indicating that retrograde superselective IACRT for tongue cancer provided excellent overall survival and locoregional control 14. Even though, poor tumor response and survival still exist in some studies. Kondo et al. achieved only 0.22 of CR rate in patients with T3 or T4 oral squamous cell carcinoma 28. The 3-year OS reported by Fujishiro et al. was 0.48 in maxillary sinus carcinoma 19. Therefore, whether IACRT has a better prognostic effect on HNC patients remains controversial. In the present study, we evaluated the efficacy of IACRT by assessing CR, 3-years OS and 5-years OS in HNC patients. The combined CR ratio, 3-year OS and 5-year OS rates were 81%, 75% and 68%, respectively, indicating that IACRT can get good control and provide high survival rates for HNC.

In clinical practice, cancers in different stages were treated by different therapies. For patients with early stage HNC, the treatment strategy is preferentially surgery. For the advanced HNC, a combined-modality treatment with surgery and radiotherapy or chemotherapy is clinically used. Therefore, we further analyzed 3-year OS rates in stage III-IV HNC and 5-years OS rates in T3-4 HNC. The 3-year OS in stage III and stage IV cancer were 75% and 52% respectively. Identically, 87% and 53% of 5-years OS were achieved in the patients with T3 and T4 HNC, respectively. A study on 201 patients with tongue SCC reported 64% 3-year OS rates in stage III and 37% in stage IV cancer by systemic chemoradiotherapy 51. Mroueh et al. compared 5-year OS of operation therapy combined with radiotherapy (OT + RT) and operation therapy combined with chemotherapy (OT + CT) among 360 patients with stage I-IV tongue cancers, and obtained a 5-year OS rate of 61% in OT + RT group, and the 69% and 51% 5-year OS rates for patients with stage III and IV in OT + CT group 52. Although these results were incomparable, the OS rate of the present study was better than radiotherapy or systemic chemotherapy. Moreover, a randomized trial on 140 HNC patients (oropharynx, maxillary antrum and intra-oral) compared the efficacy of IACRT and RT alone. The overall 5-year survival was 43% in the IACRT group and 25% in the group treated with radiation alone 53. These findings indicated that IACRT was an efficient approach to improve outcomes of HNC patients, especially for the patients with stage III or T3 cancers.

Traditional systemic chemotherapy or intravenous chemotherapy increases the concentration of drugs in blood, causing myelosuppression, leukopenia, thrombocytopenia, nausea, vomiting and damage to multiple organs 54-56. Some studies found no differences in locoregional control or overall survival between IACRT and IVCRT, but renal toxicity and late dysphagia were worse in IVCRT group comparing with IACRT 57. Kobayashi et al. compared clinical outcomes and patient's quality of life between IACRT and surgery combined with radiotherapy in patients with tongue and mouth floor SCC, IACRT showed superior to surgery plus radiotherapy in terms of the survival rate and quality of life, indicating that IACRT should be preferred in managing advanced tongue and mouth floor SCC 58. Even though, severe dermatitis and mucositis may occur within the radiation field during IACRT treatment, these toxicities were manageable and can be recovered by drug or professional oral management 59. Other studies reported that mucositis and dysphagia were regarded to be inevitable but reversible 14, 32. Additionally, systemic toxicities such as anemia, thrombocytopenia and leukopenia were also reported before 60-62. Here, we summarized ten toxicities from the 34 studies, including oral diseases, mucositis, leukopenia, dermatitis, anemia, general condition, thrombocytopenia, nausea/vomiting, fever and hepatic dysfunction. All of them were manageable and reversible. Oral diseases, mucositis, dermatitis and leukopenia were the major toxicities with relatively high incidence. Therefore, more attention should be paid to prevent these toxicities for more favorable outcomes.

The limitations of our meta-analysis should be taken into account when interpreting the results. Firstly, among the included 34 studies, only one study is RCT, due to clinically inoperable, most studies did not have control group. Therefore, the results of these studies were incomparable. Secondly, most studies were conducted in Japan, only 7 studies were conducted in other countries, this may bring both regional and cultural bias. Thirdly, the investigation sample size of some studies was small, which would increase the heterogeneity of our study.

In conclusion, the results of this study suggested that IACRT is an efficient and safe therapy for HNC with manageable toxicities, especially for advanced HNC with stage III or T3. The toxicities such as oral diseases, mucositis, leukopenia, dermatitis, general condition and anemia should be concerned during treatment. However, due to the limitations of clinical studies and the differences in IACRT methods, more high-level and large-sample clinical trials are needed to further confirm the efficacy of ICART in the comprehensive treatment for HNC.

## Figures and Tables

**Table 1:** Characteristics of enrolled studies.

Study	Year	Study design	Cancer type	Age	Male (%)	T classification	Sample size	Follow-up (y)	Country	Outcome measured
Arcangeli 18	1983	RCT	SCC	-	-	T2-T4	142	5	Italy	5-y OS
Fujishiro 19	2007	Retrospective study	Maxillary sinus carcinoma	62	78.6	T2-T4b	14	2.1	Japan	CR, 3-y OS
Furusaka 20	2012	Retrospective study	Tongue SCC	66.4[37-81]	75.6	T2-T4b	45	3.2	Japan	CR, 5-y OS
Furusaka 21	2013	Retrospective study	Hypopharyngeal piriform sinus SCC	64.1[37-74]	93	T3-T4b	57	5.3	Japan	CR, 5-y OS
Furusaka 22	2013	Retrospective study	Oropharyngeal SCC	64.6[37-86]	74.5	T2-T4b	51	5	Japan	CR,5-y OS
Fuwa 16	2008	Retrospective study	Tongue cancer	68[25-87]	65	T2-T4	40	2.3	Japan	CR,5-y OS
Fuwa 23	2008	Retrospective study	Tongue SCC	63[25-87]	71.6	T2-T4b	88	4.4	Japan	CR,3-y OS
Fuwa 24	2008	Retrospective study	OSCC	67[25-89]	66.4	T2-T4b	134	3.8	Japan	CR,3-y OS
Hayashi 25	2017	Retrospective study	OSCC	82.5[80-88]	38.7	T2-T4b	31	3.1	Japan	CR,3-y OS
Homma 26	2016	Retrospective study	Hypopharyngeal cancer	60.6[45-75]	96.7	T1-T4b	92	5.2	Japan	5-y OS
Kobayashi 27	2010	Prospective study	OSCC	61.7	63.6	T2-T4	22	1.5	Japan	CR,5-y OS
Kondo 28	2016	Retrospective study	OSCC	68[58-82]	77.8	T3, T4a	18	5	Japan	CR,3-y OS
Kovacs 15	2004	Retrospective study	Oral and oropharynx cancer.	66[38-89]	53.8	T1-T4	52	5	Germany	CR,3-y OS,5-y OS
Minamiyama 29	2017	Retrospective study	Tongue SCC	63.5[34-87]	69	T2-T4a	42	3.9	Japan	CR,3-y OS,5-y OS
Mitsudo 9	2014	Retrospective study	OSCC	59[28-87]	69.6	T2-T4b	112	3.9	Japan	CR,3-y OS,5-y OS
Mitsudo 14	2018	Retrospective study	Tongue SCC.	61[25-87]	66.1	T2-T4b	118	3.2	Japan	CR,3-y OS,5-y OS
Miyawaki 30	2012	Retrospective study	OSCC	69[39-84]	57.5	T1-T4	40	6.8	Japan	CR, 5-y OS
Mukai 31	2014	Retrospective study	Gingival carcinoma	74[50-93]	61.8	T1-T4b	34	3	Japan	CR,3-y OS,5-y OS
Takayama 32	2016	Retrospective study	Tongue SCC	53[25-69]	66.7	T2-T4a	33	3.6	Japan	CR,3-y OS
Tsukahara 33	2008	Retrospective study	Oropharyngeal SCC	63.1[44-85]	-	T1-T4	73	5	Japan	5-y OS
Yoshizaki 34	2007	Prospective study	HNSCC	61.2 ± 13.8	91.8	T2-T4	49	2	Japan	CR,3-y OS
Yoshizaki 35	2009	Retrospective study	Laryngeal Cancer	63.9	92.7	T2-T4	41	3	Japan	CR,3-y OS
Bertino 36	2007	Retrospective study	HNSCC	39-75	93.5	T2-T4	46	5	Italy	CR, 5-y OS
Bertino 37	2009	Retrospective study	HNSCC	38-74	82.1	T1-T4	56	2	Italy	CR
Doi 38	2018	Retrospective study	MSSCC	63.5[31-82]	16.7	T3-T4b	24	4.8	Japan	CR,3-y OS
Doweck 39	2008	Retrospective study	OSCC	58	81.9	T3-T4	155	-	USA	5-y OS
Hayashi 40	2019	Retrospective study	Gingival SCC	-	46.0	T2-T4b	46	3.3	Japan	CR,3-y OS
Homma 41	2013	Prospective study	MSSCC	35-74	79.6	T2-T4b	54	6.4	Japan	CR,5-y OS
Kim 42	2013	Retrospective study	Maxillary sinus carcinoma	57[32-73]	92.6	T3-T4	27	6.4	Korea	5-y OS
Krstevska 43	2012	Retrospective study	Oropharyngeal SCC		90.8	T2-T4	65	1.2	Macedonia	CR
Nakashimaa 44	2011	Retrospective study	Maxillary sinus carcinoma	37-67	60.0	T4a-T4b	5	2	Japan	CR
Nozato 45	2019	Retrospective study	OSCC	55[35-81]	61.5	T2-T4b	26	5	Japan	CR,5-y OS
Takahara 46	2017	Retrospective study	NNKTL	53[21-70]	83.3	-	12	6.8	Japan	CR
Yokoyama 47	2014	Retrospective study	Paranasal sinus cancer	66.5[45-84]	76.1	T3-T4b	46	3.1	Japan	CR
Total	34 studies						1890			

RCT: randomized controlled trial; SCC: squamous cell carcinoma; OSCC: oral squamous cell carcinoma; HNSCC: head and neck squamous cell carcinoma; MSSCC: maxillary sinus squamous cell carcinoma; NNKTL: nasal natural killer/T-cell lymphoma; CR: complete response; 3-y OS: 3-year overall survival; 5-y OS: 5-year overall survival.

**Table 2:** Analytic results of toxicities.

Toxicity	Incidence	95% CI	Number of studies Included
Oral diseases	0.515	0.266-0.765	10
Mucositis	0.589	0.417-0.762	21
Leukopenia	0.401	0.297-0.504	22
Dermatitis	0.434	0.235-0.633	14
Anemia	0.318	0.187-0.450	20
General condition	0.339	0.202-0.475	13
Thrombocytopenia	0.245	0.150-0.341	17
Nausea/vomiting	0.263	0.152-0.374	13
Fever	0.117	0.057-0.177	8
Hepatic dysfunction	0.064	0.021-0.107	7

## Abbreviations

HNC: head and neck cancer
IACRT: intro-arterial chemotherapy combined with radiotherapy
CR: complete response
OS: overall survival
CI: credible interval
IVCRT: intra-versus chemotherapy combined with radiotherapy
RCT: randomized controlled trial
SCC: squamous cell carcinoma
OSCC: oral squamous cell carcinoma
HNSCC: head and neck squamous cell carcinoma
MSSCC: maxillary sinus squamous cell carcinoma
NNKTL: nasal natural killer/T-cell lymphoma
OT: operation therapy
CT: chemotherapy
RT: radiotherapy
